# Efficacy of Dry Needling Versus Transcutaneous Electrical Nerve Stimulation in Patients With Neck Pain Due to Myofascial Trigger Points: A Randomized Controlled Trial

**DOI:** 10.7759/cureus.36473

**Published:** 2023-03-21

**Authors:** Anjana G, Anil K Gupta, Dileep Kumar, Sudhir Mishra, Ganesh Yadav, Madhumita Singha Roy, Laxmi Prajapti

**Affiliations:** 1 Department of Physical Medicine and Rehabilitation, King George's Medical University, Lucknow, IND

**Keywords:** transcutaneous electrical nerve stimulation, dry needling, neck disability index, myofascial pain syndrome, myofascial neck pain, myofascial trigger points

## Abstract

Introduction

Myofascial pain is defined as pain arising primarily in muscles and associated with multiple trigger points. Among the non-pharmacological methods, trigger point injection and electrotherapy are effective methods to treat myofascial pain syndrome. This study compares the effectiveness of dry needling (DN) and transcutaneous electrical nerve stimulation (TENS) in reducing cervical pain intensity and improving cervical range of motion in patients with neck pain due to myofascial trigger points.

Methods

Fifty patients were enrolled and randomized into two groups. Patients in group A received dry needling, and those in group B received TENS. Patients were evaluated using the Visual Analog Scale (VAS), Neck Disability Index (NDI), and Cervical Range of Motion (CROM) before the treatment and on days 14 and 28 after the treatment. The unpaired t-test was used to evaluate quantitative data, except for VAS, where the Mann-Whitney U test was used. All quantitative variables had a normal distribution with a standard deviation except for pain intensity (VAS), which deviated from the normal distribution. The significance level was set at a P-value=0.05.

Results

Both DN and TENS groups showed significant improvement in VAS, NDI, and CROM between days 0 and 28 (p=<0.001). The DN group showed greater improvements in pain intensity from day 0 to day 28 (p =<0.001). Between days 0 and 28, there was no discernible difference in NDI changes between the groups (p = 0.157 and p = 0.799, respectively). Mixed results were obtained for CROM, with significant improvement of cervical flexion in the dry needling group (p=<0.008) and significant improvement of cervical rotation to the painful side in the TENS group (<0.001).

Conclusion

Both dry needling and TENS are effective in reducing pain and improving NDI and CROM in patients with neck pain due to myofascial trigger points. However, as dry needling is more effective in pain reduction, a single session of dry needling is more beneficial and cost-effective as compared to multiple sessions of TENS.

## Introduction

Nonspecific neck pain (NSNP) is a common painful cervical spine disorder that affects 30%-50% of the general population, with about 10% of these patients experiencing chronic neck pain [1]. Some chronic neck pain is related to the myofascial system, in which there is local muscle fiber contraction that is referred to as Myofascial Trigger Points (MTrp) [2]. MTrPs can be found in all muscle groups, but they are found to be more prevalent in the upper quarter postural muscles, especially the upper trapezius muscle [3,4]. When the pain is primarily in muscles and associated with multiple tender trigger points, this condition is referred to as Myofascial pain syndrome (MPS) [5]. Among individuals with regional pain syndrome, the prevalence of MPS ranges from 21% to 85% [6]. The prevalence also increases with age, with a slight predilection for adult women [7]. Etiologically, a trigger point is a cluster of electrically active loci that is associated with a contraction knot and a dysfunctional end plate. The two types of trigger points based on clinical characteristics are “Active” (spontaneously painful; “jump sign is pathognomonic”) and “latent” (no spontaneous pain, causing only a local hypersensitivity, but may restrict movements) [8]. Other types are: associated, attachment, central, key, primary, and satellite trigger points. The pathophysiology of MTrP has been thought to be due to histological (shortened sarcomeres and tissue hypoxia) and biochemical (excessive release of acetylcholine, substance P, and lowered pH) changes, which influence the process of sensitization of the central and peripheral nervous systems [9]. Clinically, a trigger point (TrP) is identified as a localized spot of tenderness in a nodule within a palpable taut band of muscle fibers. Muscles with active myofascial TrPs have a restricted passive range of motion because of pain [10]. There are no specific biochemical, electromyographic, or diagnostic-imaging criteria granted for the diagnosis of myofascial TrPs. Manual palpation skills and patient feedback have primarily been used for TrP diagnosis and treatment [4]. Myofascial neck pain affects daily activities and functions, thus affecting the quality of life and leading to a financial burden on the healthcare system [11]. So, it is important to diagnose and treat the condition early. The main purposes of treating MPS are to eliminate pain and provide muscle strength with a full range of motion to perform activities of daily life [12]. Among trigger point injections, local anesthetic injection and dry needling are most common, but some studies suggest that dry needling can be preferred to local anesthetic as it has fewer side effects, its therapeutic effect has been correlated with the mechanical damaging of trigger points caused by needling over most tender points, and as it does not require any drugs, it is cost-effective as well [13]. Electrotherapy is another non-pharmacological, noninvasive modality useful in managing MPS. They are two major types: transcutaneous electrical nerve stimulation (TENS) and electrical muscle stimulation (EMS). TENS seems to be more effective immediately after treatment, but in long-term evaluation, there is no significant superiority between the two electrotherapy techniques [14]. The most important mechanism of action of TENS is the gate-control theory of pain control and increasing endogenous opioid release. Conventional TENS application is advantageous in reducing pain and improving the range of motion in MPS patients [12]. The trapezius muscle, which is involved in the mobility and stability of the neck and shoulder girdle, is of great importance in performing activities of daily living. MTrPs in the trapezius can cause local pain that disturbs neck and shoulder girdle functions. So it is important to diagnose and treat MTrPs in neck muscles. Many studies have been done comparing the efficacy of dry needling with other treatment modalities for MTrPs [15]. But, to the best of our knowledge, only limited studies have compared the efficacy of dry needling and TENS. However, no research has compared DN and TENS in the treatment of MTrPs in patients with neck pain. In this study, we compared the efficacy of dry needling versus TENS modalities for the treatment of myofascial trigger points of neck muscles in patients with neck pain.

## Materials and methods

**Figure 1:**
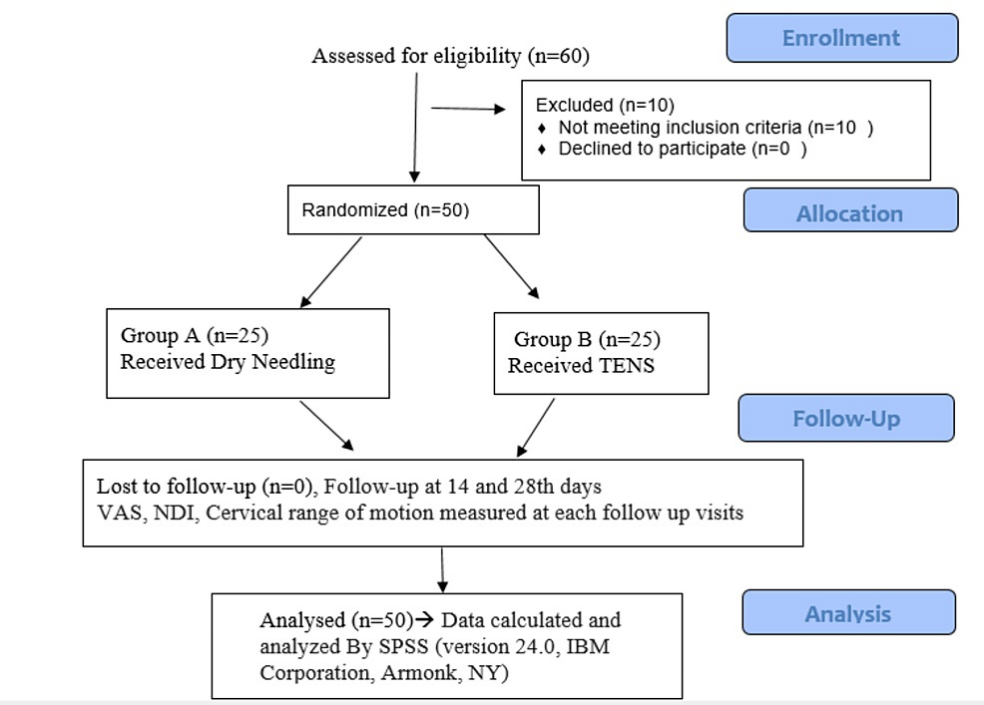
CONSORT diagram CONSORT: Consolidated Standards of Reporting Trials; VAS: Visual Analog Scale; NDI: Neck Disability Index; TENS: Transcutaneous Electrical Nerve Stimulation

This study aimed to compare the effectiveness of dry needling and TENS in treating neck pain due to myofascial trigger points of the upper trapezius. This randomized controlled clinical trial was done at the Department of Physical Medicine and Rehabilitation in a tertiary care center in northern India. The period of the study was from 20/07/2021 to 28/11/2022. A total of 50 subjects with neck pain who had at least one active trigger point (TrP) were recruited from our OPD. All the subjects received information about the purpose and examination involved in this study, and all the subjects signed written informed consent before enrollment in the study. All of the subjects were thoroughly examined. Subjects were randomized into two groups by using online software (stat trek random number generator table) [16]. Allocation concealment was done. Blinding was not feasible as the patient knew the treatment, of which one intervention was injectable. But the outcome measure was assessed by another examiner who was unaware of the treatment the patients had received, thereby reducing the information bias. The patients in the first group received dry needling, whereas the patients in the second group received TENS therapy. All participants were advised to avoid NSAIDs for three days prior to the procedure. The study was conducted according to the Consolidated Standards of Reporting Trials (CONSORT) guidelines (Figure 1).

Ethical clearance and trial registration

Patient enrollment began after approval by the institutional ethical committee (ref. no. 107th ECM IIB THESIS/P6). The study is registered with a clinical trial registry under the number CTRI/2021/07/034855.

Inclusion criteria

Consenting patients with neck pain of at least one week duration, aged between 19 and 50 years, and having at least one active trigger point were included in the study.

Exclusion criteria

Patients having fibromyalgia [17], having received similar treatment modalities within the past six weeks, include: acute trauma, inflammatory joint or muscle disease, infection, malignancy, evidence of neurological deficit, seizure disorders, cervical radiculopathy or myelopathy, Thoracic outlet syndrome, neck and shoulder surgery, pregnancy, and patients with a cardiac pacemaker.

Sample size calculation

The sample size was calculated according to a similar study conducted by Santiago et al. [18]. To achieve 90% power and a confidence level of 95% with an alpha level of p ≤ 0.05 as significant and assuming a dropout rate of 10%, 50 subjects were examined in the current study (25 per group).

Methodology

Patients with myofascial neck pain were randomized into two groups, each consisting of 25 patients. The group A received a single session of dry needling, and the group B received seven sessions of TENS therapy.

Outcome measurement

Patients were evaluated using a VAS (Visual Analog Scale), NDI (Neck Disability Index) score, and cervical range of motion measurement using the goniometry method before and at follow-up after the procedures. The VAS scale is a subjective, validated assessment scale for acute and chronic pain. Scores are measured on a 10-cm VAS scale line and are graded on a scale of 0 (no pain) to 10 (worst pain) [19]. The Neck Disability Index is a self-reported questionnaire with 10 items, including pain, personal care, lifting, reading, headaches, concentration, work, driving, sleeping, and recreation. The points range from 0 (no disability) to 50 (maximum degree of disability) [20]. The degree of cervical range of motion was assessed by clinical goniometry [21].

Intervention

The diagnosis of the trigger point was made according to Travell and Simon’s criteria [22]. Subjects in Group A, with 25 patients, received a single session of dry needling, which was done according to the Travell and Simons method [22], wherein the subject was in a sitting position, trigger point areas on neck muscles were fully determined by palpation (pincer and flat), and the trigger point was immobilized between the thumb and index finger. A 22G, 3.8-cm (1.5-inch) needle was inserted perpendicularly through the skin and moved forward until the trigger point was reached. The trigger point was identified by getting a local twitch response or contraction of the band with pain. The same point was inserted a few times with fan-shaped syringe movements. After needling, compression over the point of needling was done with cotton wool for 90 seconds [23].

Group B, with 25 subjects, received TENS therapy, which was applied by a dual-channel portable machine with symmetric bi-phasic rectangular pulses with 100 micro sec duration and a current frequency of 60 Hz. The negative electrode was placed on the active myofascial trigger point of the neck muscle, and a positive one on the appropriate tendon insertion site. The total duration of the application was 20 mins per session, with a total of 14 sessions consisting of one session per day for two weeks. Sessions were given at the hospital.

DN group patients were informed that they might experience post-needling pain and were instructed to apply ice fomentation every two hours for the first 48 hours. All subjects were telephoned 48 hours post-intervention to ensure that no adverse events occurred.

All patients were provided with a home exercise program consisting of active range-of-motion exercises for the neck and stretching exercises for the neck and back muscles. The program consisted of three sessions, and each session consisted of a repetition of every motion 20 times [24].

Follow-up and data analysis

Patients were followed up on the 14th and 28th days following the interventions, and their VAS, NDI index score, and cervical range of motion by goniometry were observed at each follow-up visit. IBM Corp. Released 2016. IBM SPSS Statistics for Windows, Version 24.0. Armonk, NY: IBM Corp. was used to examine the data. For the descriptive analysis of quantitative variables, the mean and standard deviation were used. The normality of the data was assessed by the Kolmogorov-Smirnov test. For differences between groups in NDI and cervical ROM, the unpaired t-test was used, while for pain intensity (VAS), the Mann-Whitney U test was carried out, and the significance level was set at P-value=0.05.

## Results

**Figure 2:**
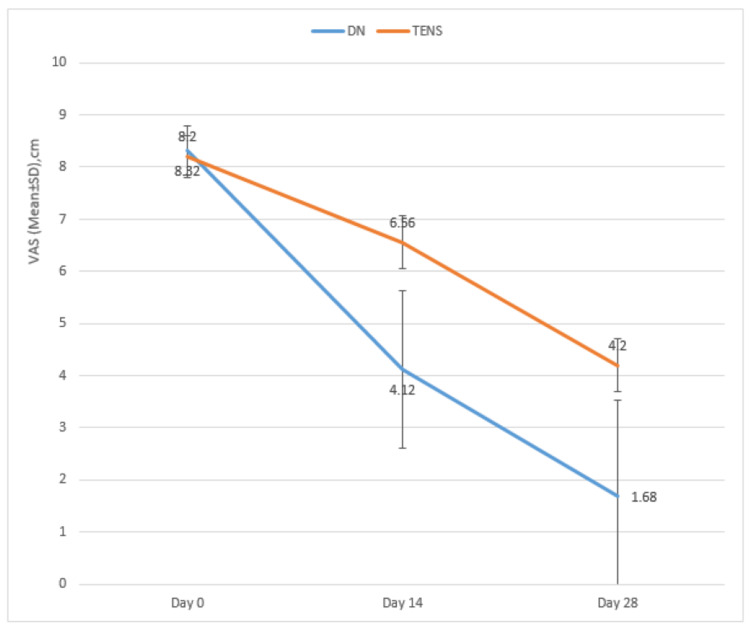
Intergroup and intragroup comparison of VAS VAS: Visual analog scale; DN: Dry needling, TENS: Transcutaneous electrical nerve stimulation. This figure shows improvement in both DN and TENS groups at follow-up, more with DN for longer duration.

**Figure 3:**
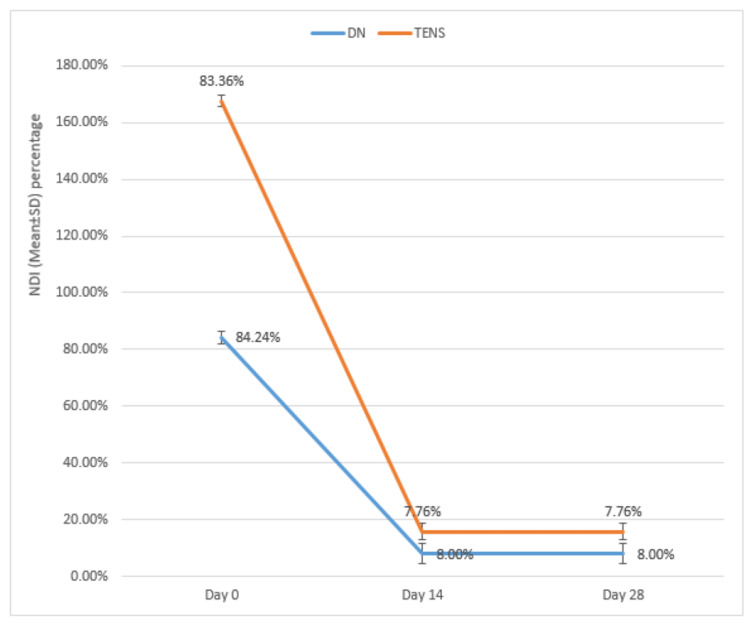
Intergroup and intragroup comparison of NDI NDI: Neck Disability Index, DN: Dry needling, TENS: Transcutaneous electrical nerve stimulation.

The mean age of the DN group was 29.16±7.12 years, while the mean age of the TENS group was 25.68±5.80 years. No significant difference was found in mean ages between the groups (p=0.064). The female-male ratio in the DN group was 52.0:48.0, while in the TENS group, the ratio was 56.0:44.0. No significant difference was found in the male-female proportion between the groups (p=0.777). The VAS of the DN group and the TENS group were compared on days 0, 14, and 28. A significant difference was found in mean VAS between the groups on days 14 and 28 (Figure 2, Table 1). The NDIs of the DN group and the TENS group were compared on days 0, 14, and 28. No significant difference was found in the mean NDI between the groups on days 0, 14, and 28, although a significant improvement was noted in both groups at all follow-ups (Figure 3, Table 2). The cervical range of motion of the DN group and TENS groups were compared on days 0, 14, and 28. An unpaired t-test found no significant differences in baseline cervical ROM measurements (i.e., cervical flexion, extension, lateral flexion, and rotation to the painful and non-painful sides) between the two groups. There were significant differences among the groups in relation to the changes (increase) in cervical ROM at all follow-ups when compared with baseline (Table 3). Unpaired t-tests revealed that there was a larger increase in cervical flexion in the TENS group compared with the DN group after the 14^th^ day (p= 0.008). The cervical extension was unaffected pre- and post-procedure. More significant improvement of painful side cervical rotation was observed in the TENS group as compared to the DN group after the 28^th^ day (p=<0.001). No significant difference was noted between the groups for painful and non-painful side cervical lateral flexion.

**Table 1:** Intergroup and intragroup comparison of VAS VAS: Visual analog scale; DN: Dry needling; TENS: Transcutaneous electrical nerve stimulation; SD: standard deviation. P<0.05 is significant.

VAS	DN		TENS		Mann-Whitney U Test	
	Mean	SD	Mean	SD	z-value	p-value
Pre-Procedure	8.32	.48	8.20	.41	-0.958	0.338
14^th^ day	4.12	1.51	6.56	.51	-5.447	<0.001
28^th^ day	1.68	1.86	4.20	.50	-4.397	<0.001

**Table 2:** Intergroup and intragroup comparison of NDI NDI: Neck disability index; DN: Dry needling; TENS: Transcutaneous electrical nerve stimulation; SD: standard deviation. P<0.05 is significant.

NDI	Group A		Group B		unpaired t-test	
	Mean	SD	Mean	SD	t-value	p-value
Pre-Procedure	84.24%	2.18%	83.36%	2.14%	1.439	0.157
14^th^ day	8.00%	3.56%	7.76%	2.91%	0.261	0.799
28^th^ day	8.00%	3.56%	7.76%	2.91%	0.261	0.799

**Table 3:** Intergroup and intragroup comparison of Cervical range of motion DN: Dry needling, TENS: Transcutaneous electrical nerve stimulation SD: standard deviation. P<0.005 is considered significant.

Cervical Range of Motion	DN		TENS		unpaired t-test	
	Mean	SD	Mean	SD	t-value	p-value
Cervical flexion day 0	58.80	3.32	57.60	4.36	1.095	0.279
Cervical flexion day 14	80.00	.00	77.60	4.36	2.753	0.008
Cervical flexion day 28	80.00	.00	79.20	2.77	1.445	0.115
Cervical extension day 0	50.00	.00	50.00	.00	0	1.000
Cervical extension day 14	50.00	.00	50.00	.00	0	1.000
Cervical extension day 28	50.00	.00	50.00	.00	0	1.000
Painful side cervical rotation day 0	63.00	4.33	63.40	4.27	-0.329	0.743
Painful side cervical rotation day 14	72.20	2.53	71.20	2.18	1.496	0.141
Painful side cervical rotation day 28	72.20	2.53	77.40	2.55	-7.234	<0.001
Non-Painful side cervical rotation day 0	54.60	4.77	56.60	5.35	-1.396	0.169
Non-Painful side cervical rotation day 14	72.20	3.25	71.60	2.38	0.744	0.460
Non-Painful side cervical rotation day 28	76.80	4.05	78.00	2.50	-1.260	0.214
Painful side cervical lateral flexion day 0	34.40	4.86	34.80	5.10	-0.284	0.778
Painful side cervical lateral flexion day 14	45.00	.00	45.00	.00	NA	NA
Painful side cervical lateral flexion day 28	45.00	.00	45.00	.00	NA	NA
Non-Painful side cervical lateral flexion day 0	31.40	3.96	30.00	5.00	1.098	0.278
Non-Painful side cervical lateral flexion day 14	45.00	.00	45.00	.00	NA	NA
Non-Painful side cervical lateral flexion day 28	45.00	.00	45.00	.00	NA	NA

## Discussion

With increasing stress and many other factors, the prevalence and clinical complications related to nonspecific neck pain are on the rise, especially in the working population [25]. There are often other coexisting pathologies in this population, which makes the diagnosis of nonspecific neck pain even more challenging. In our study, we aimed to investigate the difference between the efficiencies of dry needling (DN) and transcutaneous electrical nerve stimulation (TENS) on pain, cervical range of motion, and neck disability in patients with myofascial pain syndrome affecting the upper trapezius muscle. 

Effect on disability as measured by the neck disability index

Our study reports a significant improvement in the neck disability index (p<0.001) in both groups at all follow-ups without a significant difference between the groups. This was in contrast to the study conducted by Santiago Garcia-de-Miguel et al. [18], which showed a greater improvement of NDI in TENS as compared to dry needling. A possible explanation for these findings, as given by the author, was that it could have been due to a decrease in muscle mechanosensitivity, which would have caused the subject to feel less pain during active movements, which contract the neck muscles, thus decreasing the perception of neck disability in the TENS group. However, the reduced improvement in the dry needling group may be due to an increased dose or post-needling pain that would have masked the improvement in the neck disability index. A study conducted by Leon Hernandez et al. [26] demonstrated that there were similar effects on improvement in degree of neck disability in both TENS + dry needling and dry needling alone at 72-hour follow-up, which is consistent with our current study, which also demonstrated improvement in disability with similar effects shown by both groups. 

Effect on pain

We noted significant improvement in pain in both groups at all follow-ups, and a statistically significant difference between the groups was observed, with the DN group showing better improvement as compared to the TENS group (p<0.001). This was in contrast to the study conducted by Santiago Garcia-de-Miguel et al. [18], who noted a statistically significant difference in pain reduction in both groups (DN and TENS) without differences between the groups. This could be because of early follow-up at 48 hours and one week post-treatment, and it may be needed to allow more follow-up time to yield an appropriate difference between electrotherapy modality and dry needling. Leon Hernandez et al. [26] reported that the addition of TENS to DN significantly reduced cervical pain at post-treatment follow-up in comparison to that with DN alone. But the difference was not measured at the 72-hour follow-up. This difference between groups during post-treatment follow-up contrasts with the results of the present study, which show improvement in pain in both groups. This difference may be due to differences in TENS doses, DN technique, and its dose applied, i.e., DN that elicits more LTR results in a greater mechanical stimulus, which adds to the therapeutic potential of DN without exaggerating post-needling soreness.

Effect on Cervical Range of Motion: Our study revealed significant improvement in cervical flexion at all the follow-ups, with a statistically significant difference between the groups by day 14 (p<0.008), wherein the dry needling group showed better improvement. However, by day 28, both groups revealed similar improvements. This contrasted with the study conducted by Santiago Garcia-de-Miguel et al. [18], in which the TENS group showed greater improvement in cervical flexion as compared to the dry needling group at all follow-ups. As stated by S. Garcia-de-Miguel et al. [18], cervical flexion has the potential to increase mechanical stress on the nervous system in comparison to cervical extension. Therefore, TENS, with its action on the nervous system, would, by gate control theory and opioid-mediated analgesia, be the reason for improvement in cervical flexion. Dry needling directly acts on muscle by causing mechanical deactivation of TrPs, thereby normalizing the biochemical milieu of the involved muscle and thus improving the normal functioning of the muscle and the range of motion, and this could be the reason for the marked improvement in cervical flexion after dry needling. Improvements in cervical range of motion, such as lateral flexion and rotation, were observed in both groups, with no significant difference between them. This was similar to the study of Santiago Garcia-de-Miguel et al. [18], according to which raw values of lateral flexion and cervical rotation were within the normal range at all follow-ups in both groups, thereby making it hard to conclude about the dominance of one treatment over the other. Also, in their study, PENS (Percutaneous Electrical Nerve Stimulation) showed greater improvement in cervical lateral flexion towards both the painful and non-painful sides, whereas dry needling showed only improvement in lateral flexion towards the non-painful side. But in the present study, significant improvement (p<0.001) was noted in both painful and non-painful sides in both groups at all follow-ups. A significant difference (p<0.001) was noted between groups by day 28 post-procedure, with more improvement in painful side cervical rotation in group B. This result is almost similar to the previous study, and the reason for the better improvement noted in the TENS group could be due to its central action via gate control theory producing an anti-hyperalgesic effect. Less improvement noted in cervical side bending towards the painful side in the dry needling group can be due to the contraction of the trapezius on cervical side bending.

Limitations

One of the limitations of this study was that participants were not blinded. As a result, there could be patient-related bias. However, patients were not given choices for selecting their treatment group. Moreover, the assessor was blinded to minimize the bias. The role of comorbidities on the outcome was not assessed in this study. In this study, we assessed and treated only the upper trapezius muscle, even though other muscles can also be involved in myofascial neck pain. A control (placebo) group to further assess the efficacy of dry needling and TENS was not included in our study. A sample size with only short-term follow-up was done in our study.

## Conclusions

Both dry needling and TENS are very effective methods to improve pain, neck disability index, and cervical range of motion in patients with myofascial neck pain. Both interventions have very good safety profiles and patient compliance. Dry needling took the upper hand in improving pain for ROM, but mixed results were obtained, with TENS having the upper hand in improving cervical flexion and cervical rotation towards the painful side. As dry needling is more effective in VAS reduction, a single session of dry needling is more beneficial and cost-effective as compared to multiple sessions of TENS; however, further studies with larger sample sizes and long-term follow-up are necessary to find and confirm the long-term efficiency of dry needling and TENS in myofascial neck pain.
